# Hemoglobin E, malaria and natural selection

**DOI:** 10.1093/emph/eoz034

**Published:** 2019-12-13

**Authors:** Jiwoo Ha, Ryan Martinson, Sage K Iwamoto, Akihiro Nishi

**Affiliations:** 1 Division of Biotechnology, College of Life Sciences and Biotechnology, Korea University, Seoul 02841, Korea; 2 Department of Ecology and Evolutionary Biology, University of California, Los Angeles, CA 90025, USA; 3 College of Letters & Science, University of California Berkeley, Berkeley, CA 94720-2930, USA; 4 Department of Epidemiology, UCLA Fielding School of Public Health, Los Angeles, CA 90095, USA

**Keywords:** hemoglobin E, malaria, natural selection, malaria hypothesis

## Abstract

It is known that there has been positive natural selection for hemoglobin S and C in humans despite negative health effects, due to its role in malaria resistance. However, it is not well understood, if there has been natural selection for hemoglobin E (HbE), which is a common variant in Southeast Asia. Therefore, we reviewed previous studies and discussed the potential role of natural selection in the prevalence of HbE. Our review shows that *in vitro* studies, evolutionary genetics studies and epidemiologic studies largely support an involvement of natural selection in the evolution of HbE and a protective role of HbE against malaria infection. However, the evidence is inconsistent, provided from different regions, and insufficient to perform an aggregated analysis such as a meta-analysis. In addition, few candidate gene, genome-wide association or epistasis studies, which have been made possible with the use of big data in the post-genomic era, have investigated HbE. The biological pathways linking HbE and malaria infection have not yet been fully elucidated. Therefore, further research is necessary before it can be concluded that there was positive natural selection for HbE due to protection against malaria.

Lay summary: Our review shows that evidence largely supports an involvement of natural selection in the evolution of HbE and a protective role of HbE against malaria. However, the evidence is not consistent. Further research is necessary before it is concluded.

## INTRODUCTION

### Malaria and hemoglobin

The evolutionary connection between malaria and hemoglobin (Hb) variants was proposed in the 1940s by J.B.S. Haldane [1] and is called the ‘malaria hypothesis’ [2]. It posits that certain polymorphisms in the human genome have been selected to confer resistance against malaria [3]. After the hypothesis was proposed, a large body of epidemiology and biology studies reported an association of hemoglobin variants with malaria infection [3–6]. Moreover, genetic approaches such as haplotypic methods have been utilized to examine the hypothesis [6–8].

In 2016, 216 million people were estimated to be infected with malaria, and 445 000 people were estimated to have died from malaria in the world [9]. Southeast Asia and Africa are the most common regions for malarial infection [9]. Malaria is a life-threatening disease caused by protozoan parasites of the genus *Plasmodium*, which spread through infected anopheline mosquito [10]. There are several malarial parasite species that infect humans: *Plasmodium falciparum, Plasmodium vivax, Plasmodium malariae, Plasmodium ovale* and *Plasmodium knowlesi* [10–13]. In Southeast Asia, *P. falciparum* is the most dominant species (66%) followed by *P. vivax* (34%) [9]. In Africa, *P. falciparum* is responsible for almost all malaria cases (99.7%), whereas *P. vivax* is rare (0.3%) [9]. Symptoms of the most severe malaria infection (typically caused by *P. falciparum)* include coma, metabolic acidosis, anemia, hypoglycemia, acute renal failure and acute pulmonary edema [10]. If a severe case of malaria is left untreated, it is likely to become fatal [10]. It has been a challenge to prevent malaria infection because the parasites can avoid immune detection by expressing a different gene variant on the erythrocyte surface after the human immune system has adapted to former variants [14].

Human hemoglobin is formed by two alpha-globin and two beta-globin chains which are encoded by two α-globin genes (*HBA1 and HBA2*) and one β-globin gene (*HBB*) [15]. Hemoglobin disorders are genetic diseases affecting hemoglobin, caused by mutations and/or deletions in the α-globin or β-globin genes, and are divided into two general categories: thalassemia syndromes and structural hemoglobin variants [16]. Quantitative defects in the synthesis of α-globin or β-globin subunits, which can happen as total absences or reductions, are called thalassemia syndromes. On the other hand, qualitative disorders of globin structure are called structural hemoglobin variants [17]. These are different compared with HbA, which is the most common adult human hemoglobin [18]. Some structural variants such as hemoglobin S (HbS) and hemoglobin C (HbC) have a tendency to aggregate with sickle cell formation (the sickle cell syndromes) [19], while some other structural variants such as hemoglobin E (HbE) do not [16].

Humans have evolved several polymorphisms in human genomes and some, like glucose-6-phosphate dehydrogenase (G6PD) deficiency, are under selection by malaria to protect against malaria infection [20]. Another example is *HBB* gene variants, which exhibit balanced polymorphism, where a disadvantage of a homozygous status is offset by a heterozygous advantage against malaria [21]. Erythrocytes with such polymorphisms, including hemoglobin S and C, confer the ability to protect against malaria infection by several identified ways including the impairment of parasite growth [22–27] (see below for details).

HbS results from the switch of a glutamic acid to valine at the sixth codon of gene encoding β-globin [28]. HbC results from an alteration of a glutamic acid to lysine [15]. Multiple studies on the roles of HbS (dominant in sub-Saharan Africa and the Middle East) and HbC variants (in West Africa) in protecting against malaria have been conducted. First, impairment in parasite growth by HbS [27] and HbC erythrocytes has been observed [22, 23]. Second, HbS and HbC may slow the trafficking of parasite proteins across the parasitophorous vacuole [29]. Third, HbC and HbS carriers have been found to produce increased levels of immunoglobulin-G directed against several plasmodial antigens [30, 31]. Fourth, the microRNA of HbAS and HbSS red blood cells (RBCs) play a protective role against malaria [32]. Fifth, one study suggests an interaction between HbC and genes regarding the activation of natural killer (NK) cells [33], which plays an important role in combatting human malaria [34]. Moreover, several epidemiologic studies also suggest that HbS and HbC play a role in malaria resistance [35–38]. Lastly, a genetic association study has confirmed the association with resistance to malaria and both HbS and HbC [39]. The current consensus is that these two variants, particularly HbS, support the malaria hypothesis and were favored by natural selection [3].

### Hemoglobin E

HbE is a variant caused by a single point mutation at codon 26 of the β-globin gene [40], which is located on chromosome 11p15.5 [8]. This point mutation leads to the replacement of glutamic acid (Glu: GAG) with lysine (Lys: AAG) [41], resulting in abnormal messenger RNA processing [42] and irregular erythrocytes [41]. The gene expression of the HbE mutation leads to the improved efficiency of normally inactive donor sites for RNA splicing at the codon of the β-globin site [42]. The mutation makes it resemble the consensus splice sequence ‘AAGGTGAGT’, often referred to as ‘cryptic’ [42, 43] due to it being non-detectable in wild-type pre-mRNA [44]. Therefore, the cryptic splice site becomes activated and leads to the reduced production of normally spliced mRNA as a result of aberrant splicing [45]. A relative reduction in β-globin results in the accumulation of excess α-globin chains, leading to a minor globin-chain imbalance [46]. Individuals homozygous for the HbE allele (HbEE or ‘hemoglobin E disease’) have microcytic hypochromic anemia, and heterozygotes (HbAE or ‘hemoglobin E trait’) have mild anemia [16]. People possessing an HbE variant may also develop secondary disorders like jaundice, hepatosplenomegaly and growth retardation in their developmental stages, which leads to the diagnosis of HbE [41]. The clinical severity for these patients strongly depends on whether or not they also have thalassemia [41]. For example, HbE/β-thalassemia is the most dominant combination in Asia and is also responsible for 50% of all severe β-thalassemia cases globally [41, 47]. HbE cases are most commonly found in Southeast Asia [48], and the prevalence reaches 50% in some groups in Thailand [49].

Analysis of Southeast Asian populations suggests that the HbE mutation in Thailand originated between 1240 and 4440 years ago [8]. One paper suggests that there are at least five haplotypes of the HbE mutation (three in Southeast Asia and two in Europe) [50], with other papers, including a recent study, suggesting that there are multiple independent origins in Southeast Asia [49, 51, 52]. A study confirmed that an HbE variant found in Yunnan, China shares the same origin as one found in Thailand, and also indicated that consanguineous marriage might explain the variation in the frequency of HbE [53]. Current evidence supports the rise and evolution of the HbE mutation in different locations throughout Asia [51]. There is a mechanism that explains how the HbE mutation may appear on more than one haplotype in a given population: meiotic recombination [51, 52]. For example, Jomoui *et al.* [52] suggested that two of the most common haplotypes of the β^E^-globin gene found in the Southeast Asian population could have resulted via recombination between restriction sites. In addition, other papers reported the existence of a recombination hotspot in the β-globin gene cluster [54–58].

## STUDIES ON THE ASSOCIATION BETWEEN HbE AND MALARIA

Unlike HbS, HbC and other protective polymorphisms like G6PD deficiency, the positive natural selection of HbE due to benefits offered against malaria is controversial. Therefore, we performed a literature review of HbE studies to summarize and evaluate the current evidence. We introduce three different series of studies investigating HbE and malaria: biological mechanisms of HbE, evolutionary genetics studies including a linkage disequilibrium (LD) analysis and epidemiologic studies including a genetic association study.

### Protective biological mechanisms

We have identified four major potential mechanisms explaining how HbE can protect against malaria.

First, reduced probability of parasite merozoite invasion into the RBCs can provide resistance to malaria [21]. In other words, abrogation of a specific parasite–erythrocyte binding interaction is important, and factors such as bending and stability might be major factors in the inhibition of parasite invasion whereas membrane deformability is only weakly related [59]. There are *in vitro* studies that investigate the connection between HbE and malaria resistance. Chotivanich *et al.* performed a mixed erythrocyte invasion assay and calculated the susceptibility ratio (SR), which is denoted as (stained infected RBCs/stained RBCs)/(unstained infected RBCs/unstained RBCs). Median SR of HbAA = 0.88 (range = 0.53–1.4) > Median HbEE = 0.69 (range = 0.13–0.98) > Median HbAE = 0.34 (range = 0.05–0.64) [21]. This finding suggests a protective role of HbE and was supported by other *in vitro* studies [24, 60]. On the other hand, another study compared seven HbAE patients with seven HbAA patients and found that the level of infected RBCs was not associated with the status of HbE carriers after a 96-hr incubation of RBCs and *P. falciparum* [61]. However, this sample size was much smaller than previous studies [21] such as the aforementioned study by Chotivanich *et al.* which had samples of HbAA (*n* = 34), HbAE (*n* = 20) and HbEE (*n* = 22). There are two important considerations for interpreting *in vitro* studies such as a mixed erythrocyte invasion assay. First, if the malaria strains used for the *in vitro* studies are different from those prevalent in the Southeast Asian region for the past thousands of years, they can easily produce false negative results. Second, the results of *in vitro* studies do not directly represent what occurs *in vivo*.

Second, after the entry of malaria into an erythrocyte, remodeling the host cell structure and the growth of the parasite are important for the next steps of replication and egress [62]. Therefore, one of the mechanisms of malarial resistance is the inhibition of this process [21]. Studies reported that the growth of malaria parasites is inhibited in HbE cells. The severity of acute *P. falciparum* was ameliorated in the presence of HbE [63], and other studies reported a decrease in growth of the parasite in HbE erythrocytes [24–26]. In addition, a recent study reported significantly lower *P. vivax* asexual parasite densities in HbAE heterozygotes compared with HbAA *P. vivax* patients (*P* < 0.05) [64]. However, other studies suggested that HbE does not have an inhibitory role in parasite growth [65–67].

Third, an alteration in the morphology of erythrocytes in response to parasite infection plays an important role in the removal of infected cells [68]. Erythrocytes infected with *Plasmodium* parasites showed decreased deformability [69–71], resulting in increased retention in the spleen [69, 72], which is responsible for the removal of abnormal erythrocytes [68]. One notable morphological characteristic in HbE cells is a slight microcytosis [73].

Fourth, infected HbE cells are more susceptible to phagocytosis by monocytes [60]. The surface alterations of *P. falciparum*-infected erythrocytes such as excrescences, knobs [74], new antigenic determinants [75], modification of membrane lipids [76], membrane fluidity [77] and binding to endothelial and amelanotic melanoma cells [78] were suggested by various experiments [60]. One recent study found that HbE/β-thalassemia RBCs triggered the phagocytic function of monocytes and that HbE/β-thalassemia monocytes have increased RBC clearance compared to normal monocytes, which was controlled by the upregulation of microRNA-155 [79].

Lastly, there are several other suggested protective mechanisms. For example, HbE is associated with high antimalarial antibody titers [24, 80]. In addition, redox status and function in variant cells are a determinant of infectability [81]; that is, HbE erythrocytes possess impaired antioxidant defenses [82, 83] creating an undesirable environment for oxidation-sensitive malaria parasites [84]. Finally, the improved clearance rate of the parasite by artemisinin derivatives in individuals with HbAE has been documented [85].

In sum, we have identified and summarized various biological mechanisms of HbE. However, a list of the potential mechanisms is not sufficient to discuss the involvement of natural selection in the evolution of HbE. It has not been fully examined which mechanism can make a difference in human survival and reproduction or which mechanism makes HbE advantageous in an environment.

### Evolutionary genetics studies

LD is a non-random association of certain alleles that are coinherited with a higher probability than predicted by random chance within a population, and this can be due to selection or founder effects [86, 87]. The reduction or elimination of variation near a mutation in DNA is called a selective sweep, inducing a high prevalence of certain alleles in the genomic region near the causal variant. These associated alleles define a haplotype and remain in the population until recombination separates them [86]. As LD declines due to recombination, the length of the LD segment can be used to estimate when an advantageous mutation appeared [87]. LD studies are very important to infer and analyze evolutionary history and disease inheritance [87].

Ohashi *et al.* [8] performed an LD analysis to calculate a pairwise |D’| in 48 Thai patients with mild *P. falciparum* malaria. All subjects were recruited from the Suan Phueng District in the Ratchaburi Province (a single hospital), which is 200 km west of the Bangkok metropolitan area and near the border with Myanmar. Additionally, they performed a statistical test for detecting reduced haplotype diversity and ran forward in time computer simulations under a variety of selection models [8]. The study suggests that the HbE mutation occurred between 1240 and 4440 years ago under positive selection and that the allele frequency increased rapidly [8]. The study provides the strongest evidence of natural selection for HbE. In fact, we did not find any counter evidence to this claim in all 81 studies citing the study. However, to our present knowledge, no other studies have attempted to replicate the Ohashi *et al.*’ study with other populations.

As stated in the prior section, different human populations could have developed the same or similar hemoglobin mutations despite different origins: there are five haplotypes of HbE with three Southeast Asian and two European origins [50], and at least two independent origins in Southeast Asia [51, 52]. The independent origins of HbE mutations and simultaneous increase in the frequency of HbE in various populations increase the likelihood of positive natural selection, although genetic drift cannot be ruled out by this fact alone. Compared with HbS, HbE has few origins and haplotypes due to its rapid and recent rise [8].

In sum, the evolutionary genetics studies such as an LD analysis have potential to provide strong evidence supporting natural selection for HbE. Although the Ohashi *et al.*’s study using the Thai population used a robust study design and provided clear evidence, a single study using a single population is not sufficient to draw a conclusion. A larger number of evolutionary genetics studies using various relevant populations from the malaria-affected regions must be conducted to confirm the findings of the Ohashi *et al.*’s study.

### Epidemiologic studies

Taylor *et al.* [88] showed using the data of two case-control studies [63, 89] that there was no association of HbE status with the ‘severity’ of the *P. falciparum* infection. This analysis needs to be carefully interpreted as one case study showed a strong protective effect, while the other showed a null effect. There is a large heterogeneity within the small-scale meta-analysis [88]. Given these mixed results with other outside studies [90, 91], the role of the HbE variant in malaria severity has still been controversial.

Studies examining an association of HbE status with the presence of *P. falciparum infection* (comparing malaria cases with non-malaria cases) can be more relevant. Kar *et al.* [92] reported a significantly reduced prevalence of *P. falciparum* parasitemia in HbAE/EE (4.3%) compared with AA (12.2%) (*N* = 708, northeast India) (*P* < 0.001). Their results are also partially supported by a recent study, Shannon *et al.* (*N* = 202, Bangladesh) [93] which showed that HbEE (OR 5.0 [1.07–46.93]), not HbAE (OR 0.71 [0.42–1.19]), was associated with increased passive case detection of *P. falciparum*. One recent study reported that groups with HbAE (*P* = 0.006) and HbEE (*P* = 0.004) had significantly lower odds of presenting with acute *P. vivax* infections using the HbAA group as the reference (*N* = 247, China–Myanmar border) [64]. Additionally, one study observed high prevalence of HbE in highly malarious regions compared to the regions with a low incidence of malaria [94]. Recently, one study reported high prevalence of co-inheritance of HbAE and G6PD deficiency [95], which is another variant that provides protection against malaria and has gone through positive selection [96]. In addition, Deng *et al.* pointed out that the time frame of HbE origination (between 1240 and 4440 years ago) [8] coincides with the origination of the most common G6PD variant in Southeast Asia, which had been affected by positive selection over the past 1500 years [97].

Genetic association studies, including genome-wide association studies (GWAS), are another significant way to explain host genetics and parasite interactions and genetic correlations with common diseases [98, 99]. Several genetic association analysis have been conducted [98–100] but there is a lack of data from populations, where HbE is common [39]. A study investigated the single nucleotide polymorphism (SNP) responsible for HbE, rs33950507, in S’tieng ethnic group in Vietnam and stated that the sample was too small to estimate association with severe malaria [39]. Other studies showed that some SNPs affect the severity of HbE/β^0^-thalassemia [101, 102].

Epistatic interactions, where the presence of one gene influences the phenotypic expression of another gene [103], are expected to take place between hemoglobin genes. Positive epistasis between the α- and β-thalassemia genes leading to malaria protection has been reported [104]. Elimination of protection against severe malaria by co-inheritance of HbS and α-thalassemia, negative epistasis, has also been reported and might provide an explanation for the low prevalence of α-thalassemia variants in Africa [105]. One epidemiological study suggested negative epistasis between HbF and HbS on malaria [103]. We found no studies focusing directly on epistasis between HbE and other hemoglobin disorders. One study aimed to test gene–gene interaction signals of association at pairs of SNPs that showed strong evidence of association with severe malaria [39]. However, this study was conducted without the inclusion of Thai, Cambodian, Laotian populations which have the highest HbE prevalence. In addition, it only investigated the influence of *P. falciparum*, whereas Southeast Asian populations also suffer from *P. vivax*. Future studies should investigate the influence of *P. vivax* and include more data from Southeast Asian populations.

In sum, epidemiologic studies have repeatedly shown a positive association of HbEE or HbAE with reduced malaria cases. However, the evidence is inconsistent and reported from different countries and regions. Available data is not sufficient to perform an aggregated analysis such as a standard meta-analysis and a meta-regression. Furthermore, there are variations in the study designs and the reported outcomes of the above-mentioned studies. Genetic association studies including a SNP for HbE have not been often implemented.

## CONCLUSIONS AND FUTURE PERSPECTIVES

Based on the literature review, we conclude that HbE seems to be a good candidate for fulfilling the malaria hypothesis [3, 48, 49, 106] (summarized in Table 1). Most current evidence does not reject the potential role of natural selection in the evolution of HbE, in which the selection pressure is malaria. Multiple epidemiologic studies that have shown a positive association of HbE with reduced malaria cases and conducted an LD analysis provide the strongest evidence to support natural selection for HbE. None of the studies strongly suggested an involvement of genetic drift or other evolutionary mechanisms. If positive natural selection acts to increase the allele frequency of HbE, we would see evidence for this in different types of studies. For example, LD analysis studies and other genetic studies would repeatedly detect positive selection such as a selective sweep, and epidemiologic studies would repeatedly find a reduced frequency of malaria cases among people having HbE. What we found in the literature review here is not like this. Rather, we found that the populations relevant to HbE have been understudied and this may lead to the lack of strong evidence for the potential association of HbE and malaria.

**Table 1.** Studies on the association between HbE and malaria^a^

Year	Ref no.	Results	**R** ^b^
1961	[106]	Suggested that the high prevalence of HbE in Southeast Asia is due to conferred resistance against malaria	+
1964	[94]	Higher prevalence of HbE in the highly malarious regions, compared with the regions with a low incidence of malaria were observed	+
1967	[48]	Suggested that HbE heterozygote gives protection against malaria	+
1969	[67]	HbE and thalassemia traits did not confer advantages for *P. falciparum* infection regarding infection rate and parasite densities	−
1981	[26]	HbEE showed slowed multiplication of *P. falciparum*, while the parasite grows normally in HbEA	+
1981	[65]	No differences between HbAA, HbEA and HbEE toward parasite growth were reported	−
1986	[60]	Susceptibility of *P. falciparum*-infected HbE erythrocytes to phagocytosis by monocytes is increased.	+
1986	[24]	Compared with HbAA, parasite growth diminished as the concentration of HbE increased, and it showed higher titer value of antimalarial antibody	+
1987	[82]	HbE RBCs possess impaired antioxidant defense and this impairment would limit the development of the malaria parasite.	+
1987	[25]	Decreases in the growth of *P. falciparum* in HbE/β-thalassemia patients were observed	+
1992	[92]	Malaria is an important ecologic factor for maintaining the high frequency of HbE	+
1992	[59]	Cell deformabilities of HbE/β-thalassemia and HbEE erythrocytes have little influence on parasitic invasion, but other various factors may play an important role on inhibition of parasite invasion	+
1995	[89]	There were no significant differences between mean parasitemia levels in HbAA, HbEE and HbEA	−
1999	[63]	HbE trait may reduce the severity of acute *P. falciparum*	+
2000	[85]	HbE trait may enhance the antimalarial effect of artemisinin derivatives	+
2002	[21]	HbAE red cells showed increased resistance to invasion of the parasite, although not from HbEE	+
2004	[8]	Recent origin of the HbE variant was analyzed by extended linkage disequilibrium and suggested the intense selection as a force	+
2008	[91]	For the onset of cerebral malaria, HbE polymorphism is not an important genetic factor	−
2009	[80]	Significant increase in the frequency of malaria antibodies to *P. falciparum* and *P. vivax* in HbE/β-thalassemia patients	+
2012	[88]	Meta-analysis of 2 case-control studies showed that there was no association between HbE and the severity of *P. falciparum* infection, with the significant heterogeneity	−
2014	[39]	A genetic association study was conducted but the sample size was too small to estimate the association with severe malaria	
2015	[93]	HbEE, not HbAE, was associated with increased passive case detection of *P. falciparum*	+
2015	[90]	Parasitemia with coexistence of HbE was lower than that of other thalassemia, which also showed that they had lower parasitemia than patients without coexistence of thalassemia	+
2016	[53]	Variation in the HbE frequency may be influenced by the selective pressure of malarial presence	+
2016	[61]	Despite small sample numbers, presence of HbE did not affect growth of *P. falciparum*	−
2017	[95]	The high ratio of HbE is resultant of the selection by malaria, and high prevalence of co-inheritance of HbAE and G6PD deficiency was reported	+
2017	[52]	Multiple origins of HbE gene in the Asian population have been confirmed, and natural selection is responsible for a high ratio and spread of this gene in Southeast Asia	+
2017	[79]	HbE/β-thalassemia RBCs triggered the phagocytic function of monocytes	+
2018	[64]	Significantly lower *P. vivax* asexual parasite densities in HbEA heterozygotes were observed when compared with HbAA *P. vivax* patients	+
2018	[66]	HbE does not have an inhibitory role in neither *P. falciparum* nor *P. vivax* growth	−

a There are several studies that have been conducted regarding the association between HbE and malaria. A total of 22 studies reported positive association, while negative from 7 studies and 1 for unmeasurable due to small sample size.

b Positive (+) or negative (−) relationship between HbE and malaria.

Therefore, readers should be careful regarding the interpretation. While the current research suggests that HbE is a good candidate for fulfilling the malaria hypothesis, richer data with more robust statistics are necessary before drawing a conclusion. Specifically, future research should look for more evidence of a selective sweep near the HbE mutation. Ohashi *et al.*’ study [8] only looks at a single district in Thailand, and the results have not been reproduced in other studies. If future studies show evidence of a selective sweep near HbE mutations in other Thai populations or in non-Thai populations, this will support the conclusion.

Since the ongoing 1000 Genomes Project includes two potentially relevant populations: Yunnan, China (CDX) and Ho Chi Minh, Vietnam (KHV), this can be an important data source for future HbE research [107]. Other data in Southeast Asia may be also useful [108–110]. Genome-wide Composite of Multiple Signals score, which identifies SNPs and regions that are under recent natural selection in the genome, has been provided only in US (CEU), Nigerian (YRI) and Japanese and Chinese (JPT+CHB) populations [111].

Furthermore, few genetic studies, including genetic association studies and epistasis studies, have focused on HbE or populations where HbE is common. For example, one multi-country study [39] had been conducted without including countries with a high prevalence of HbE (only Vietnam was included among the 11 selected countries; they are mostly in Africa), and this study did not consider the influence of *P. vivax,* while HbE carriers are mainly populated in the regions where both *P. vivax* and *P. falciparum* are endemic.

It appears that there is a research gap in the malaria and hemoglobin literature. One of the potential reasons why research on HbS and HbC have been well-funded and supported, while HbE is not, is that the US government set a priority on researching sickle cell disease in response to pressure by African American advocacy groups starting in 1971, which led to a large allocation of funds for studying sickle cell diseases [112]. However, HbE does not present a sickle cell phenotype and is not classified as thalassemia syndrome despite following the disease pattern of β-thalassemia [16].

Although the present article and many of the cited references focus on the association of HbE, malaria and natural selection in Southeast Asia, new trends have occurred during the past century—migration and urbanization. These may change the evolutionary consequences of HbE. First, there has been a significant Asian migration and Asian births in the USA, many of whom were HbE carriers [113]. In the State of California alone, 25% of Cambodian newborn babies and 11% of Thai or Laotian newborn babies had the HbE variant [113]. Since patients who display HbE along with other Hb mutations or thalassemias are expected to have more severe symptoms with variable health outcomes [41], it may be important to screen adults for hemoglobinopathies at an early stage [47]. Second, it is known that urbanization reduces malaria transmission owing to unsuitable environments for malaria vectors [114], a greater degree of access to health care services [115] and increased number of human population per mosquito vectors [116, 117]. Several studies have shown the impact of urbanization to *P. falciparum* [114, 115, 118, 119] and *P. vivax* [120] endemicity. These studies found lower transmission rate in urban areas. The lower risk of malarial infection in urban areas, which means that there are less benefits to having Hb variants against malarial infection.

Both migration and urbanization can alter the level of fitness of HbE in particular environments, which depends on the benefit of protection against malaria and negative health consequences of HbE. Quantifying the influence of migration and urbanization can be one of the directions of future research. Even if it is true that natural selection favors HbE in particularly malaria-rich regions and populations, this may not be the case in other regions and populations, especially in urbanized regions and migrated populations.
